# Involvement of TRPV1 Channels in Energy Homeostasis

**DOI:** 10.3389/fendo.2018.00420

**Published:** 2018-07-31

**Authors:** Stewart Christie, Gary A. Wittert, Hui Li, Amanda J. Page

**Affiliations:** ^1^Vagal Afferent Research Group, Centre for Nutrition and Gastrointestinal Disease, Adelaide Medical School, University of Adelaide, Adelaide, SA, Australia; ^2^Nutrition and Metabolism, South Australian Health and Medical Research Institute, Adelaide, SA, Australia

**Keywords:** TRPV1, appetite regulation, metabolism, obesity, endovanilloid, endocannabinoid

## Abstract

The ion channel TRPV1 is involved in a wide range of processes including nociception, thermosensation and, more recently discovered, energy homeostasis. Tightly controlling energy homeostasis is important to maintain a healthy body weight, or to aid in weight loss by expending more energy than energy intake. TRPV1 may be involved in energy homeostasis, both in the control of food intake and energy expenditure. In the periphery, it is possible that TRPV1 can impact on appetite through control of appetite hormone levels or via modulation of gastrointestinal vagal afferent signaling. Further, TRPV1 may increase energy expenditure via heat production. Dietary supplementation with TRPV1 agonists, such as capsaicin, has yielded conflicting results with some studies indicating a reduction in food intake and increase in energy expenditure, and other studies indicating the converse. Nonetheless, it is increasingly apparent that TRPV1 may be dysregulated in obesity and contributing to the development of this disease. The mechanisms behind this dysregulation are currently unknown but interactions with other systems, such as the endocannabinoid systems, could be altered and therefore play a role in this dysregulation. Further, TRPV1 channels appear to be involved in pancreatic insulin secretion. Therefore, given its plausible involvement in regulation of energy and glucose homeostasis and its dysregulation in obesity, TRPV1 may be a target for weight loss therapy and diabetes. However, further research is required too fully elucidate TRPV1s role in these processes. The review provides an overview of current knowledge in this field and potential areas for development.

## Introduction

Obesity has become the fifth leading cause of death, and the second leading cause of preventable death worldwide, closely following tobacco smoking (1, 2). There are multiple hormonal, neurotransmitter, and receptor systems involved in the regulation of energy balance. Pharmacological attempts to favorably modulate these systems to encourage weight loss have been somewhat effective, although not without adverse side effects. This has led to the search for more suitable targets. One such group of receptors/ion channels gaining attention for their possible role in energy homeostasis are the Transient Receptor Potential (TRP) channels.

TRP channels are a superfamily of about 28 non-selective cation channels divided into 7 subfamilies including TRP vanilloid (TRPV), and TRP ankyrin (TRPA) (3). They were first identified in 1969 from an irregular electroretinogram in a mutant strain of the *Drosophila* fly (4). The electroretinogram presented a short increase in retinal potential which gave rise to the name “transient receptor potential” (5). Since their discovery, TRP channels have been identified as osmo- and mechano-sensitive (6). For example, TRPA1 is associated with pain sensations and inflammation (7), and TRPV1 is associated with pain and temperature regulation (8).

Endotherms use energy to create heat to maintain body temperature and in colder climates it has been shown that humans expend more energy for thermoregulation compared to warmer climates (9). Given the high energy costs of generating heat to maintain an optimal cellular environment thermoregulation can also play an important role in energy homeostasis. TRPV1 channels are involved in thermoregulation, making them a possible target for the modulation of energy expenditure. Further, it is becoming apparent that TRPV1 may be involved in the regulation of appetite via the modulation of appetite hormones and/or by acting on gastrointestinal vagal afferents. This is a process that may involve interaction with the endocannabinoid system considering that endocannabinoids such as anandamide (AEA), produced in the gastrointestinal tract are also endogenous TRPV1 agonists. In addition, there are suggestions that TRPV1 may be involved in the regulation of insulin secretion in the pancreas. Studies in obese individuals have suggested that TRPV1 may be dysfunctional or dysregulated due to loss of effect on energy homeostasis. For this reason TRPV1 may be a potential target for pharmacological manipulation to aid in weight loss with recent studies suggesting selective blockade or activation of specific functions of TRPV1. However, due to its complexity this may prove difficult. This review explores TRPV1 structure and modulation and will focus on its involvement in energy homeostasis, diabetes, and possible pharmacological manipulation.

## TRPV1 channels

TPRV1, the first channel in the vanilloid family, is highly permeable to calcium and was discovered in 1997 by cloning dorsal root ganglia expressed genes in human embryonic kidney cells (10). It is expressed in a wide range of central and peripheral tissues. Centrally, TRPV1 is highly expressed in the brain stem, mid-brain, hypothalamus and limbic system (11). Peripherally it is expressed in many tissues including the vagal and spinal sensory nerves (12), stomach (13), and adipose tissue (14).

### TRPV1 structure

**Figure 1:**
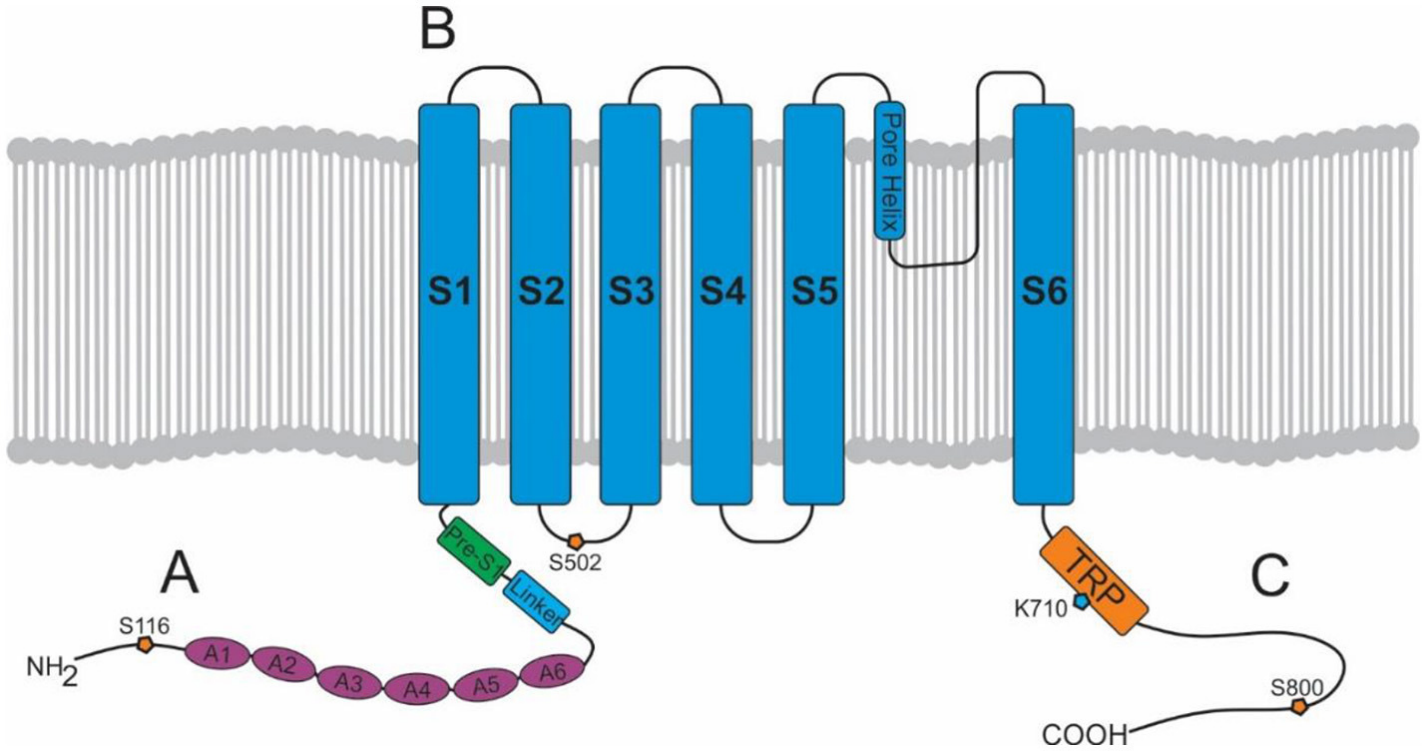
Structure of a TRPV1 subunit. **(A)** N-terminus containing 6 ankyrin subunits (A1–A6) and a linking region consisting of a linker and a Pre-S1 helix segment. **(B)** Transmembrane region with 6 helical segments (S1–S6). **(C)** C-terminus containing a TRP domain and binding sites for PKA, PKC, PIP_2_, and calmodulin.

The TRPV1 channel consists of four identical subunits located in the plasma membrane with each subunit (Figure 1) consisting of an N-terminus, a transmembrane region, and a C-terminus (15, 16). The N-terminus contains an ankyrin repeating domain consisting of 6 ankyrin subunits (16) which in its tertiary structure forms six α-helices connected by finger loops (15). Sites on the N-terminus are capable of phosphorylation by protein kinases with the S116 phosphorylation site being one of functionality (17). A linker section connects the N-terminus to the transmembrane region via the pre-helical segment (pre-S1), and connects TPRV1 subunits together (15–18).

The transmembrane region of each TRPV1 subunit comprises 6 helical segments (S1–S6), where S1–S4 contribute to the voltage-sensing domain, and S5–S6 contribute to the pore-forming domain (16). S1-S4 are connected to S5-S6 by a linker segment, and act as a foundation which allows the linker segment to move, contributing to pore opening and TRPV1 activation. The transmembrane region also contains binding sites for several ligands. For example, vanilloids (e.g., capsaicin) are capable of binding to S3 and S4, and protons (H^+^) are capable of binding to S5 and the S5–S6 linker (pore helix) (15).

Lastly, the C-terminus consists of a TRP domain (TRP-D) which interacts with pre-S1 suggesting a structural role (16). Following the TRP domain are several protein kinase A (PKA) and protein kinase C (PKC) phosphorylation sites, and sites for binding calmodulin and phosphatidylinositol-4,5-bisphosphate (PIP_2_) (15, 16).

### TRPV1 channel activation or modulation

TRPV1 is activated by a wide variety of different stimuli including heat, protons (*pH* < 5.9) (8, 19), capsaicin the irritant compound in hot chilies (10), allicin and diallyl sulfides from garlic (20, 21), peperine from black pepper (22), and gingerol from ginger (23). Spider and jellyfish venom-derived toxins are also TRPV1 agonists (24, 25).

Endogenous agonists are referred to as endovanilloids. To qualify as an endovanilloid the compound should be produced and released in sufficient amount to evoke a TRPV1-mediated response by direct binding and subsequent activation of the channel. Further, to permit regulation of the channel the signal should have a short half-life. Therefore, the mechanisms for synthesis and breakdown of the endovanilloid should be in close proximity to TRPV1. As the binding sites for endogenous ligands of TRPV1 are intracellular (26, 27) then the ligand could also be produced within the cell or there should be a mechanism to bring it into the cell. Three different classes of lipid are known to activate TRPV1 i.e., N-acyl-ethanolamines [NAEs, e.g., AEA (28)], some lipoxygenase products of arachidonic acid and N-acyl-dopamines (e.g., N-arachidonoyldopamine, N-oleoyldopamine) (29). Further, adipose tissue B lymphocytes (B1 cells) that regulate local inflammatory responses produce leukotrienes including leukotriene B4 which is also a TRPV1 agonist (30).

Intracellularly, calmodulin, a calcium-binding messenger, mediates the negative feedback loop formed by calcium (31). Calcium binds and activates calmodulin allowing it to bind to the N-terminus or C-terminus of TRPV1 inhibiting TRPV1 activity (15). Other secondary messengers such as PKA, PKC, and PIP_2_ are also capable of modulating TRPV1 activity. PKA can enhance or activate TRPV1 through phosphorylation of sites (S116 and T370) on the N-terminal (32) and may play a role in the capsaicin induced Ca^2+^ dependent desensitization of TRPV1 activation, a phenomenon which has been extensively reviewed elsewhere (33). PKC directly activates TRPV1 through phosphorylation of the S2–S3 linker region (S502) and C-terminal sites (S800), and also potentiates the effect of other ligands such as protons (34, 35). PIP_2_ is a negative regulator, inhibiting TRPV1 activity when bound to the C-terminal sites (TRP domain: K710) (36).

### Interactions between TRPV1 and the endocannabinoid system

#### The endocannabinoid system

The endocannabinoid system consists of endocannabinoids, their receptors and the enzymes involved in endocannabinoid synthesis and degradation. This system is involved in many physiological processes including memory, mood, and relevant to this review promotion of food intake (37). Endocannabinoids are endogenous lipid messengers (e.g., AEA and 2-arachydonoyl-glycerol) which activate their receptors, cannabinoid receptor-1 (CB1) and cannabinoid receptor-2 (CB2) (38, 39).

**Figure 2:**
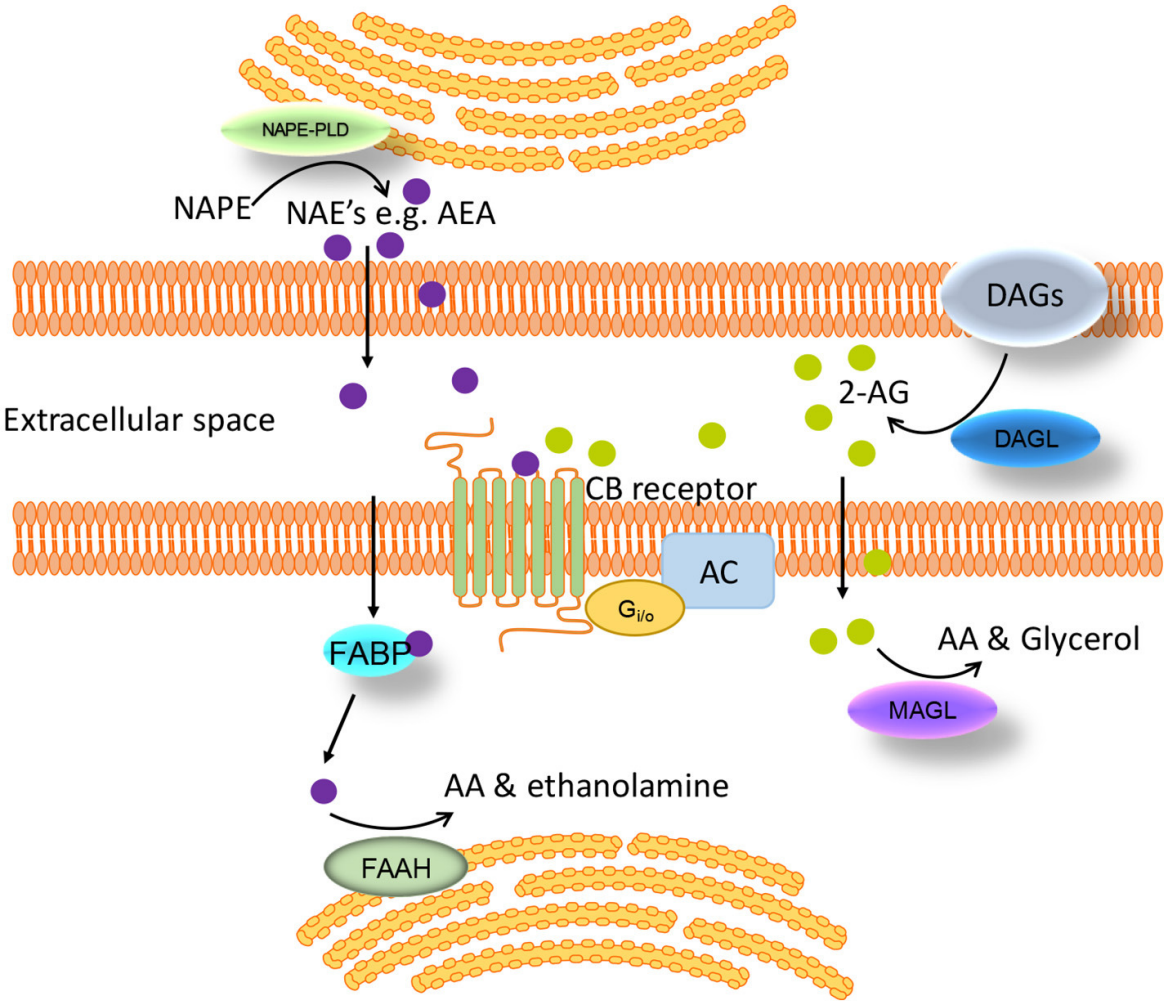
Schematic of the synthesis, degradation and action of endocannabinoids at cannabinoid receptors. Endogenous lipid messengers, such as AEA and 2-AG, act on cannabinoid receptors. AEA and 2-AG are synthesized on demand and degraded by cellular uptake and enzymatic hydrolysis by FAAH and MAGL respectively. FABP carries AEA from the cell membrane to the endoplasmic reticulum where it is finally converted to AA by FAAH. AA, arachidonic acid; AC, adenylate cyclase; AEA, anandamide; 2-AG, 2-arachidonoylglycerol; CB, cannabinoid; DAGs, diacylglycerols; DAGL, diacylglycerol lipase; FAAH, fatty acid amide hydrolase; FABP, fatty acid-binding protein; MAGL, monoacylglycerol lipase; NAE, *N*-acylethanolamines; NAPE, *N-*acylphosphatidylethanolamine; NAPE-PLD, NAPE-specific phospholipase D.

These endogenous lipid messengers are synthesized on demand and degraded by cellular uptake and enzymatic hydrolysis [see review (40) and Figure 2]. Briefly, the first step in the synthesis of AEA and NAEs is the transacylation of membrane phosphatidyethanolamine-containing phospholipids to N-acylphosphatidyl-ethanolamines (NAPEs) (41, 42). There are a number of ways that NAPEs are metabolized to their corresponding NAE including catalyzed hydrolysis by the NAPE-hydrolysing enzyme phospholipase D (NAPE-PLD) (43). In contrast, diacylglycerol lipase (DAGL) is responsible for the formation of 2-AG (44). There is still some controversy on whether there is an endocannabinoid membrane transporter [See reviews (45, 46)]. Nonetheless, endocannabinoids can be cleared from the extracellular space. Further, there are intracellular proteins that can shuttle these lipids to specific intracellular locations (e.g., TRPV1 for AEA) (47, 48), the best characterized of these are the fatty acid-binding proteins (FABP e.g., FABP5 and 7) (49). The enzymes responsible for the breakdown of AEA and NAEs are fatty acid amide hydrolase (FAAH) and N-acylethanolamine-hydrolysing acid-amidase (NAAA). NAAA is predominantly located in the lungs where it is localized to the lysosomes of macrophages (50, 51). FAAH is more ubiquitous and FAAH-1 is located on the endoplasmic reticulum whereas FAAH-2 (not found in rodents) is located in the lipid rafts (52, 53). Monoacylglycerol lipase (MAGL) is the enzyme responsible for the majority of 2-AG hydrolysis in most tissues (54–56).

The receptors for endocannabinoids, CB1 and CB2 are members of the G-protein coupled receptor family, being predominantly coupled to the G_i/o_α proteins that inhibit adenylyl cyclase thereby reducing cellular cAMP levels (57, 58). However, coupling to other effector proteins has also been reported, including activation of G_q_ and G_s_ proteins, inhibition of voltage-gated calcium channels, activation of inwardly rectifying potassium channels, β-arrestin recruitment and activation of mitogen-activated protein kinase (MAPK) signaling pathways (59). As a result of CB receptor signaling through multiple effector proteins the probability of biased signaling (ligand-dependent selectivity for specific signal transduction pathways) increases. Biased signaling is thought to occur when different ligands bind to the receptor causing different conformational changes to the receptor enabling the receptor to preferentially signal one pathway over the other (60, 61). This is attractive, in terms of development of pharmacotherapies for various diseases, as it suggests the possibility of being able to design a drug that will activate/inhibit a specific intracellular pathway.

The endocannabinoid system drives food intake via CB1 (62). Administration of CB1 agonists induces feeding in rodents (63) and humans (64), while blocking CB1 reduces food intake (65). Further, overactivity of the endocannabinoid system perpetuates the problems associated with obesity (62) and drugs targeting CB1 have been used therapeutically to manage obesity but withdrawn due to CNS side effects (66). New evidence indicates the endocannabinoid system can control food intake by a peripheral mechanism of action (66). Peripherally-restricted CB1 antagonists, with no direct central effects, reduce food intake and body weight in rodents (67, 68).

#### The endocannabinoid system and TRPV1

**Figure 3:**
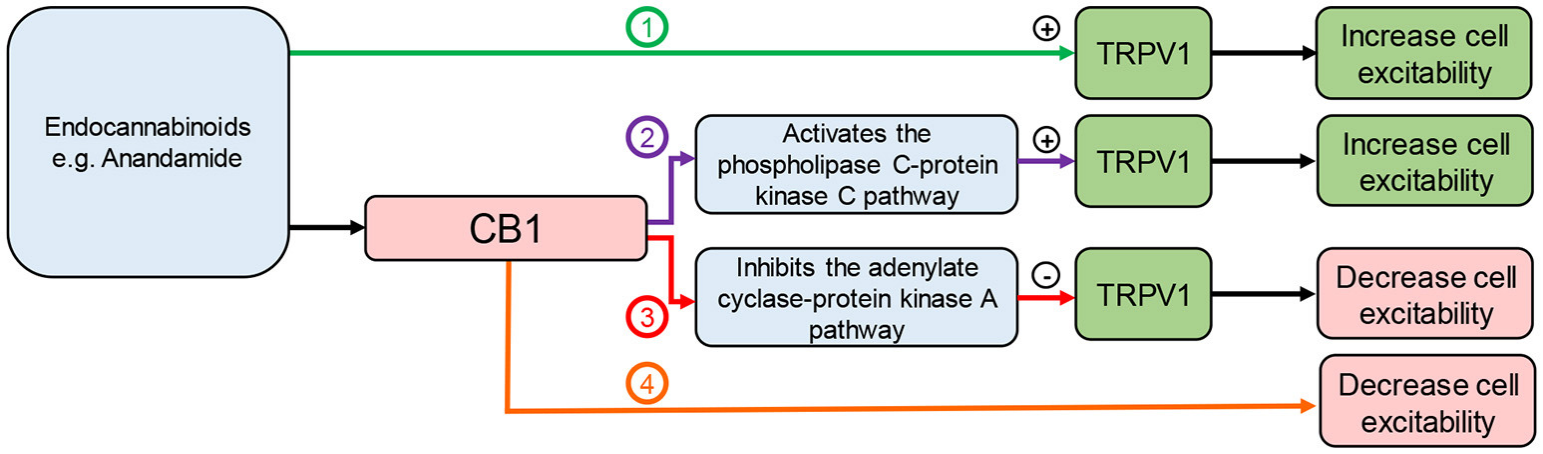
Interaction between endocannabinoids, TRPV1, and CB1. Endocannabinoids can: (1) directly activate TRPV1 leading to cannabinoid receptor 1 (CB1)-independent effects; (2) activate CB1 leading to activation of the phospholipase C pathway enhancing TRPV1 activity; (3) activate CB1 leading to inhibition of the adenylate cyclase pathway inhibiting TRPV1 activity; (4) activate CB1 leading to TRPV1-independent effects.

Endocannabinoids, such as AEA, are also endogenous ligands for TRPV1 (69). Capsaicin, an agonist of TRPV1, has an anti-obesity effect in rodents (14) and reduces food intake in humans (70). Therefore, the effects of endocannabinoids on food intake will depend on the site of action (Figure 3). This is complicated further as effects may be mediated via cross talk between TRPV1 and CB1. It has been demonstrated that CB1 can enhance or inhibit TRPV1 channel activity depending on whether it activates the phospholipase C (PLC)-PKC or inhibits the adenylate cyclase (AC)-PKA pathways respectively (71) (Figure 3). This interaction appears to be dose-dependent. Moderate to high concentrations of AEA (1–10 μM) have been shown to activate TRPV1 in a PKC dependent manner (34, 35). Conversely, low doses of AEA (3–30 nM) inhibit TRPV1 activity (72, 73), presumably through CB1 mediated inhibition of AC (74). Therefore, enzymatic synthesis and breakdown of endocannabinoids are potentially important determinants of TRPV1 activity in tissue, such as neuronal tissue, that co-express TRPV1 and CB1 (71). A clearer understanding of the role endocannabinoids play in food intake regulation in health and obesity is required to determine the physiological relevance of these different interactions. In mice, the levels of AEA in the small intestinal mucosa and plasma were elevated in high fat diet-induced obese mice compared to controls (68) but still within the low dose range (3–30 nM) shown to inhibit TRPV1 (72, 73). Consistent with these observations food intake was reduced by treatment with a peripherally restricted CB1 antagonist (68). Similar observations were made in humans with plasma anandamide levels elevated in obese compared to overweight or lean individuals, again to levels consistent with TRPV1 inhibition. Therefore, the physiological significance of TRPV1 activation observed at moderate to high concentrations of AEA remain to be determined.

## Involvement of TRPV1 in energy homeostasis

Reports on TRPV1 mediated regulation of energy intake and expenditure are conflicting. Nonetheless, epidemiological data indicate that consumption of food containing capsaicin is associated with a lower prevalence of obesity (75, 76). Further, in a clinical trial capsinoid supplementation for 12 weeks decreased body weight in overweight individuals compared to the placebo control group (77). In a separate trial, capsinoid supplementation for only 4 weeks resulted in a trend toward a decrease in body weight (78). Under laboratory conditions, dietary capsaicin supplementation had no effect on body weight (14) in mice fed a standard laboratory diet. However, in high-fat diet-induced obese mice, dietary supplementation of capsaicin significantly reduced weight gain (14, 79, 80). Further, reduced weight gain was also observed in high fat diet mice after topical application of capsaicin (81). This appears to be consistent across species as a study in rabbits, fed a standard laboratory diet supplemented with cholesterol and corn oil, demonstrated that dietary capsaicin reduced weight gain (82). In contrast, it has been shown that a 6 week dietary capsaicin treatment had no effect on body weight in high fat diet mice (83). Weight gain in TRPV1-knockout (KO) mice has been reported to be reduced (84), increased (85) or unchanged (86, 87) compared to wild type mice. This variability may reflect the study design. For example, TRPV1 channels can be activated by the endogenous ligand AEA (69). The production of AEA is dependent on dietary fat and therefore even slight changes in diet will impact on research outcomes. A summary of the effect of TRPV1 on energy homeostasis in humans can be found in Table 1. The following sections integrate the data on energy intake and expenditure from human and animal studies in an attempt to draw some clear conclusions and directions for further study.

**Table 1:** Effects of Capsaicin Supplementation on food intake and metabolism in Humans.

**Capsaicin dosage**	**Duration**	**Appetite effects**	**Metabolic effects**			**References**
			**Energy expenditure**	**RQ value**	**Blood glucose**	
Capsaicin (7.68 mg/day)	36 h	↓ Energy intake trend ↑ Satiety	–	–	–	(88)
Chili (1.03 g/meal)	24 h	↑ Satiety	↑ Thermogenesis	–	–	(89)
Capsaicin (7.68 mg/meal)	24 h	No effects	↑ Energy expenditure ↑ Fat oxidation	↓	–	(90)
Chili (1 g/meal)	1 meal	↓ Energy intake ↑ Satiety trend	↑ Energy expenditure	↓	–	(91)
Chili (0.3 g/meal)	5 meals	No effects	–	–	–	(92)
Chili (1.03 g/meal)	1 meal	↑ Plasma GLP-1 ↓ Plasma ghrelin trend	No effect	–	–	(93)
Capsaicin 26.6 mg	1 meal	–	–	–	↓	(94)
Capsaicin + green tea	3 weeks	↓ Energy intake ↑ Satiety	–	–	–	(95)
Chili 3 g + caffeine 200 mg	24 h	↓ Energy intake ↓ Fat intake	↑ Energy expenditure ↑ SNS activity	–	–	(70)
Capsaicin 150 mg	1 meal	–	↑ Fat oxidation	↓	–	(96)
Chili 0.9 g/meal	2 days	↓ Energy intake ↑ Satiety	–	–	–	(97)
Chili with meal	1 meal	↓ Energy intake trend ↓ Fat intake	–	–	–	(98)
Capsaicin 3.5 mg with glucose drink	1 meal	–	–	–	↓	(99)
Capsaicin 135 mg/day	3 months	↓ Plasma leptin (likely due to weight loss)	↑ Fat oxidation	↓	↓	(100)
Capsaicin 3 mg/meal	1 meal	–	↑ Energy expenditure ↑ SNS activity effects lost in obesity	–	–	(101)
Chili 6 g in appetizer	1 meal	↓ Energy intake ↓ Carbohydrate intake	↑ SNS activity	–	–	(102)
Chili 10 g/meal	1 meal	↓ Energy intake trend ↓ Protein and fat Intake	↑ Thermogenesis ↑ Fat oxidation	↓	–	(102)
Chili 10 g before meal	1 meal	–	No effect	↑	No effect	(103)
Chili 10 g/meal	1 meal	–	No effect	↑	–	(104)

### Role of TRPV1 in energy intake

The effects of capsaicin supplementation on satiety and food intake are illustrated in Table 1. In human studies, dietary supplementation of a TRPV1 agonist such as capsaicin, or the less pungent sweet form capsiate, caused a short-term trend or significant decrease in energy intake along with an increase in satiety (88, 89, 91, 92, 97). These effects could at least in part be due to the effect of TRPV1 on appetite hormones and/or gastrointestinal vagal afferents. This will be discussed in detail below.

Conversely, other data from human (90, 92) and animal studies (82, 105–108) suggest that dietary supplementation of capsaicin has no effects on energy intake. This could be due to capsaicin mediated TRPV1 desensitization where food intake is initially reduced, due to capsaicin activation of the TRPV1 channel, but shortly returns to normal, due to a desensitization of the channel following the initial transient activation (14). In a Chinese adult cohort study, it has been shown that energy intake depends on the amount of chili consumed with individuals with chili consumption below 20 g per day and above 50 mg per day having reduced and increased energy intake respectively, compared to non-consumers (76). Therefore, it is possible that at low levels of consumption capsaicin activates TRPV1 leading to a reduction in food intake and at high levels it could be desensitizing TRPV1 leading to an increase in food intake. However, this is highly speculative and requires further investigation.

Dietary supplementation of capsaicin can also influence nutrient preference. It has been demonstrated that capsaicin ingestion reduced the desire for and subsequent intake of fatty foods (91, 97, 98), whilst also increasing the desire for and intake of carbohydrates (92, 97). Conversely, in other studies, capsaicin ingestion reduced the desire for and consumption of carbohydrates, and increased the desire for salt rich foods (91, 102). The sensory mechanisms responsible for the changes in food preferences remain to be determined.

#### TRPV1 and appetite hormones

There is evidence to indicate that TPRV1 interacts with appetite regulating hormones, most notably, ghrelin, leptin, and glucagon-like peptide-1 (GLP-1). Ghrelin is an orexigenic peptide mainly expressed in the stomach as an endogenous ligand for the growth hormone secretagogue receptor (GHSR) (109). It is involved in many processes including appetite regulation, secretion of gastric acid, gastrointestinal motility, and regulation of energy storage (110). It has been reported that TRPV1 activation reduced plasma ghrelin levels (93), which may account for the reduced food intake observed after capsaicin supplementation (88, 89, 91, 92, 97). However, this requires more intensive investigation. Within the stomach ghrelin maybe involved in the interaction between the endocannabinoid system and TRPV1. CB1 receptors co-expressed with ghrelin in specialized cells within the stomach wall (111). Ghrelin reduces gastric vagal afferent mechanosensitivity, in a manner dependent on nutritional status, via action at GHSR expressed on vagal afferents (112–115). Therefore, although the eating stimulatory effects of ghrelin are not thought to be mediated by vagal afferents (113), ghrelin acting on vagal afferents may impact on the amount of food consumed after the initiation of a meal. Inhibition of CB1 decreases gastric ghrelin secretion with subsequent, vagal afferent mediated, reductions in food intake (111). Therefore, part of the effect of endocannabinoids on vagal afferent activity maybe mediated indirectly via the activation of CB1 on ghrelin-producing cells. It is conceivable that the inhibitory effects of ghrelin are mediated via TRPV1 considering that, in the CNS, ghrelin effects on supraoptic magnocellular neurons are mediated via TRPV1 (116). Similar, interactions with the endocannabinoid system are observed centrally in areas associated with appetite regulation, including the hypothalamic arcuate and paraventricular nuclei (117). A comprehensive investigation of the interactions between the endocannabinoid system, ghrelin and TRPV1 is required to fully understand their role in appetite regulation.

Leptin is a satiety hormone produced and secreted in proportion to the amount of white adipose tissue (WAT). Data suggests that it is also secreted by gastric cells (118). There is evidence to suggest that TRPV1 and leptin may interact, since TRPV1 -/- mice exhibit increased basal leptin levels, even when normalized to WAT mass (85). Exogenous administration of leptin normally results in decreased food intake; however, this was not observed in TPRV1 -/- mice (85). Furthermore, there is evidence for direct interactions between leptin and TRPV1 in certain brain stem regions. For example, TRPV1 activation increased the frequency of miniature excitatory synaptic currents in leptin receptor containing neurons of gastric-related dorsal motor nucleus of the vagus (DMV) (119). These data suggest that TRPV1 may mediate the effects of leptin; however, further research is needed to substantiate these claims and to determine if leptin effects in the periphery are also mediated through TRPV1.

GLP-1 is a peptide hormone secreted by intestinal L-cells, pancreatic α-cells, and neurons in the brainstem and hypothalamus (120). Evidence suggests it is involved in appetite regulation, gastric emptying, gastrointestinal motility (121), insulin secretion, and glucagon inhibition (122). Capsaicin supplementation enhanced the increase in plasma GLP-1 levels observed after a meal (93) suggesting TRPV1 channel activation may play a role in GLP-1 secretion. This requires further investigation but has the potential to be a peripheral target for the treatment of obesity and/or diabetes.

#### TRPV1 and gastrointestinal vagal afferents

Gastrointestinal vagal afferents are an important link between the gut and brain. They relay information on the arrival, amount and nutrient composition of a meal to the hindbrain where it is processed and gastrointestinal reflexes are coordinated with behavioral responses and sensations such as satiety and fullness (123–125). The role of gastrointestinal vagal afferents in the control of food intake has been extensively reviewed previously (126). Briefly, as food is ingested the vagal afferents innervating the stomach respond to mechanical stimulation as undigested food enters, fills and distends the stomach wall. There are two fundamental classes of mechanosensitive vagal afferent ending in the stomach according to location and response to mechanical stimulation (127, 128): mucosal receptors respond to fine tactile stimulation and tension receptors respond to distension and contraction of the stomach wall. Gastric mechanosensitive vagal afferents can be modulated by gut hormones and adipokines in a nutritional status dependent manner (114, 129, 130). As gastric emptying occurs, nutrients enter the small intestine and interact with nutrient receptors on the surface of specialized cells within the intestinal mucosa. This initiates an intracellular cascade that culminates in the release of gut hormones (126). These hormones can act in a paracrine fashion on vagal afferent endings innervating the small intestine and/or act as true hormones by coordinating activities within the gut or by entering the circulation and acting in the brain.

It has been demonstrated that TRPV1 is expressed in rat duodenal (131), mouse jejunal (132) and mouse gastric vagal afferents (13, 87). Activation of TRPV1, by oleoylethanolamide (OEA), caused depolarisation of nodose neurons and decreased short-term food intake (133). Further, OEA increased gastric vagal afferent tension receptor mechanosensitivity in lean but not high fat diet-induced obese mice (87). In standard laboratory diet fed TRPV1^−/−^ mice, the response of gastric vagal afferent tension (but not mucosal) receptors to mechanical stimulation was reduced compared to TRPV1^+/+^ mice (13, 87). This was associated with an increase in food intake in the standard laboratory diet fed TRPV1^−/−^ mice (87). However, the increase in food intake could also be due to the involvement of TRPV1 in gut hormone release (93, 97) or its interaction with leptin in central regions, such as the DMV (119), as described in detail above. Nonetheless, this data suggests that TRPV1 is involved in gastric vagal afferent signaling.

In high fat diet-induced obese mice the response of gastric tension receptors to distension was dampened (87) an effect also observed in jejunal vagal afferents (134). Gastric tension receptor mechanosensitivity in high fat diet-fed TRPV1^−/−^ mice was not significantly different compared to standard laboratory diet fed TRPV1^−/−^ mice (87). This suggests that disrupted TRPV1 signaling plays a role in the dampened vagal afferent signaling observed in high fat diet-induced obesity, however, this requires further investigation. Interestingly, CB1 receptors are also expressed in vagal afferent neurons (135, 136) and therefore it is conceivable that there is an interaction between TRPV1 and CB1 in gastric vagal afferent signaling, however, this has yet to be confirmed.

### Role of TRPV1 in energy expenditure

There is increasing evidence that capsaicin ingestion may have desirable metabolic outcomes such as increased metabolic rate and fat oxidation. It was reported that dietary capsaicin supplementation lowered the respiratory quotient indicating decreased carbohydrate oxidation and increased fat oxidation (90, 91, 96, 100). In contrast, there is data demonstrating that dietary capsaicin increased the respiratory quotient (104, 103). The differences in study design, which may account for the different outcomes, include method of ingestion (capsule vs. meal), active ingredient (capsinoid vs. capsaicin) and the population studied (habitual chili consumers, non-habitual, normal weight, overweight, fitness level). For example, the study by Lim et al. specifically used “runners” for their investigation (103). There is some evidence that capsaicin can elevate energy expenditure by action on the sympathetic nervous system (SNS) or adipose tissue; this is discussed below.

#### TRPV1 and the sympathetic nervous system

The SNS is involved in many processes and is probably best known for its involvement in the “flight or fight” response. Dietary supplementation of capsaicin increases postprandial SNS activity (70, 101, 102). Capsaicin excites TRPV1 containing afferent nerves, carrying a signal to the spinal cord (137). Efferent nerves are then excited by the central nervous system leading to elevated catecholamine (e.g., epinephrine, norepinephrine, and dopamine) release from the adrenal medulla (137–139). Catecholamines can bind β-adrenergic receptors increasing expenditure and thermogenic activity (104, 140). This suggests that TRPV1 may directly stimulate heat production. Further, these effects of TRPV1 on SNS activity are lost in obese subjects suggesting TRPV1 dysfunction in obesity (101).

#### TRPV1 and adipose tissue

Adipose tissue plays a key role in energy homeostasis (141). WAT generally stores excess energy as lipids, and oxidizes these stores when required, whereas, brown adipose tissue (BAT) is specialized for energy dissipation (142, 143). TRPV1 has been shown to be expressed in 3T3-L1 and HB2 adipocyte cell lines, brown adipocytes, BAT and WAT (144–147). Data indicate that TRPV1 may prevent the development of mature adipocytes from pre-adipocytes, and decrease their lipid content by increasing lipolysis (14). This may partially explain the decreased lipid accumulation during dietary supplementation of capsaicin. Further, it has been demonstrated that capsaicin induces browning in differentiating 3T3-L1 preadipocytes (145). Therefore, TRPV1 could be involved in the browning of WAT and the thermogenic activity of brown adipose tissue (BAT). The levels of TRPV1 mRNA in BAT and WAT are reduced in HFD-induced obesity and leptin receptor deficient mice (147) suggesting possible involvement in the development of obesity.

**Figure 4:**
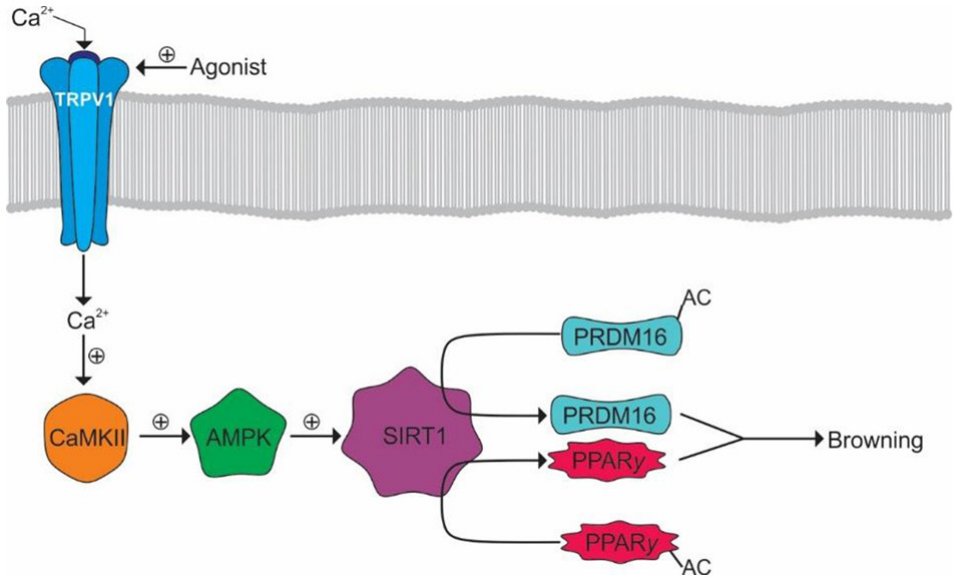
Browning of WAT by TRPV1 activation. Activation of TRPV1 results in Ca^2+^ influx and the subsequent activation of CaMKII. CaMKII facilitates the subsequent activation of AMPK and SIRT1 allowing the deacetylation of PRDM16 and PPARγ allowing their interaction and promotion of WAT browning.

Browning is a process whereby WAT becomes thermogenic in nature, similar to BAT. The calcium influx from TRPV1 activation may mediate this process by activating the peroxisome proliferator-activated receptor gamma (PPARγ) and positive regulatory domain containing 16 (PRDM16) pathways (107). Calcium binds and activates calmodulin-dependent protein kinase II (CaMKII) leading to the subsequent activation of adenosine monophosphate activated protein kinase (AMPK) and sirtuin-1 (SIRT-1). SIRT-1 deacetylates PRDM and PPARγ causing browning events such as thermogenesis (Figure 4) (107).

**Figure 5:**
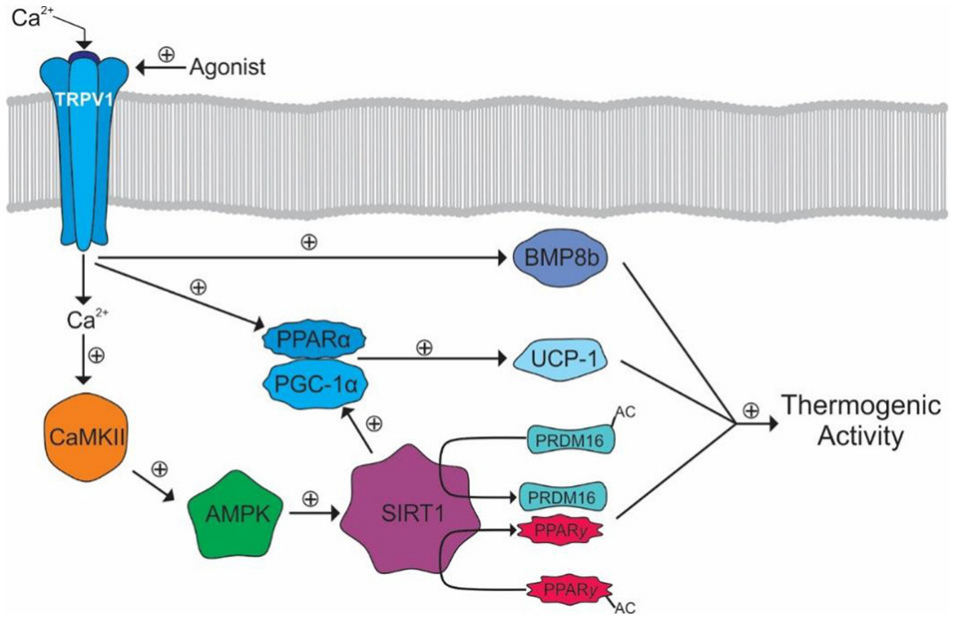
TRPV1 mediated activation of thermogenic activity in BAT. The calcium influx activates CaMKII leading to the eventual activation of SIRT1 and deacetylation of PRDM16 and PPARγ. SIRT1 also activates PGC-1α leading to the activation of PPARα. PGC-1α and PPARα transcriptionally activate UCP-1. TRPV1 also activates BMP8b which, along with UCP-1, PRDM, and PPARγ, cause increased thermogenic activity.

TRPV1 activation may also promote BAT thermogenesis either through modulation of the SNS or via direct activation of BAT. However, research in this area is limited. The PPARγ and PRDM16 pathway, previously mentioned in WAT, has been shown to be activated by TRPV1, via SIRT1, in BAT (107, 108). Further, SIRT1 also activates peroxisome proliferator-activated receptor gamma coactivator 1-α (PGC-1α). PGC-1α transcriptionally activates PPARα subsequently leading to the production of uncoupling protein-1 (UCP-1) (108). UCP-1 is a mitochondrial protein that uncouples the respiratory chain triggering a more efficient substrate oxidation and thus heat generation (148). Lastly, TRPV1 activates bone morphogenic protein 8b in BAT, which also contributes to thermogenesis (Figure 5) (108).

Regulation of BAT by TRPV1 can also be via an indirect mechanism through modulation of the SNS. Activation of TRPV1 in the gastrointestinal tract, by capsaicin or its analogs, has been shown to enhance thermogenesis and activate UCP-1 in BAT in mice (149, 150) via a mechanism mediated via extrinsic nerves innervating the gastrointestinal tract (149). This is consistent with reports that TRPV1 ligands (capsaicin and acid) increase gastrointestinal afferent activity (151, 152) via TRPV1 (152). Further, it has been demonstrated that ingestion of capsinoids increases energy expenditure through activation of BAT in humans (153). Gastrointestinal vagal afferents have central endings in the nucleus tractus solitaries (NTS). The NTS has projections to BAT (154) where it regulates the sympathetic tone to BAT (155) and has been directly implicated in the control of thermogenesis (156, 157). Lipid activation of duodenal vagal afferents has been shown to increase BAT temperature via a cholecystokinin dependent mechanism (158). In contrast, it has been reported that vagal afferent activation decreases BAT sympathetic nerve activity and BAT thermogenesis in rats (159). Further, glucagon-like peptide-1 activation of gastrointestinal vagal afferents leads to a reduction in energy expenditure and BAT thermogenesis in mice (160). It is possible that different subtypes of gastrointestinal vagal afferent have different roles in the control of BAT thermogenesis, however, this requires further investigation along with the role of TRPV1 in this gut-brain-BAT pathway.

Capsaicin can also evoke a heat-loss response which could conceivably result in compensatory thermogenesis to maintain thermal homeostasis. Capsaicin evoked complex heat-loss responses have been shown in various mammals including the rat, mouse, guinea-pig, rabbit, dog, goat, and humans (161). In humans, cutaneous vasodilation and sweating in response to hot chili consumption is well recognized (161). In the rat it has been demonstrated that capsaicin elicited cutaneous vasodilation resulting in a reduction in core body temperature (162). Simultaneously, capsaicin also enhanced heat production (162). In these experiments, it appeared that capsaicin independently activated pathways for heat production and heat loss and therefore the observed thermogenesis may not be a simple compensatory mechanism in response to heat loss, however, this requires further investigation.

## Involvement of TRPV1 in diabetes

### Type 1 diabetes

Insulin is a hormone, secreted by β-cells of the pancreatic islets, which regulates blood glucose levels. Type 1 diabetes is an autoimmune disease involving T cell-targeted destruction of pancreatic β-cells. TRPV1 is expressed in sensory nerves innervating the pancreas. Chemical denervation of these TRPV1 containing pancreatic afferents, using high doses of capsaicin (approximately 150 mg/kg body weight), significantly reduced blood glucose levels and increased plasma insulin (163), suggesting that TRPV1 containing pancreatic afferents negatively regulate insulin secretion. Further, chemical destruction of TRPV1 expressing neurons in neonatal mice (164) was able to protect the mice from autoimmune diabetes (165). Chemical denervation of TRPV1 containing pancreatic afferents significantly reduced the levels of pre-type 1 diabetes immune markers such as CD4^+^ and CD25^+^ T-regulatory cells in pancreatic lymph tissue and reduced the infiltration of CD8-CD69 positive effector T-cells (165, 166); immune cells implicated in the destruction of pancreatic islets in type 1 diabetes. However, as the afferents are destroyed and the treatment is not selective for TRPV1, it is plausible that the observed effects have nothing to do with TRPV1. Pancreatic islets also include resident dendritic cells which are generally believed to express TRPV1 (167–169) although there is some controversy (170). Activation of TRPV1 channels on dentritic cells could activate cell function including antigen presentation to CD4^+^ T cells. Further, TRPV1 channels have been shown to be expressed on rat pancreatic β-cells where they control the release of insulin leading to reduced blood glucose levels (171) and capsaicin has been shown to reduce blood glucose by increasing insulin levels in a streptozotocin-induced diabetic rat model (172). Taken together, these data suggest that TRPV1 may influence insulin secretion and type 1 diabetes acting via a number of different cell types within the pancreas.

### Type 2 diabetes

Insulin resistance, closely linked to obesity (173), occurs when cells are less responsive to insulin. As a consequence there is reduced blood glucose uptake leading to increased blood glucose levels. Pancreatic β-cells normally respond to this by increasing output of insulin to meet the needs of the tissues. Development of type 2 diabetes stems from a failure of the β-cells to adequately compensate for insulin resistance (174). It has been demonstrated that postprandial insulin levels were lower after the consumption of a standardized meal seasoned with cayenne pepper (175). As the plasma glucose levels were not significantly different from the control group the authors suggested that glucose clearance occurred similarly with lower levels of insulin, implying increased insulin sensitivity after the consumption of the hot meal. Further, consumption of chili has been shown to decrease postprandial insulin levels in obese subjects (176). In support of these studies, TRPV1^−/−^ mice have been shown to be more insulin resistant than wild type mice (85).

Type 2 diabetes is believed to be associated with inflammation (177, 178). It is believed that inflammation in the pancreas leads to an increase in the activity of TRPV1 which contributes to increasing levels of calcitonin gene-related peptide (CGRP) (179). CGRP is known to promote insulin resistance and obesity by decreasing insulin release from β-cells (180).

## TRPV1 as a pharmacological target for obesity and diabetes

Behavioral interventions (e.g., diet and exercise) alone are seldom sufficient for the intervention of obesity and diabetes. Combining behavioral and pharmacological approaches is becoming increasingly more attractive. However, pharmacological interventions can have hard to access targets and/or adverse side effects. TRPV1 is present in the periphery making it an easily accessible target compared to drugs that target the central nervous system. However, TRPV1 interacts with other systems and shares pathways commonly used by other signaling molecules. Therefore, without a clear understanding of the interactions of TRPV1 with other systems, the targeting TRPV1 for the treatment of obesity and diabetes is unlikely to be successful, as evident from the numerous contradictory studies looking at the effect of capsaicin analogs on food intake and weight gain. Data suggest that manipulation of TRPV1 may be possible in such a way to reduce or eliminate any unwanted side effects. For example, three different TRPV1 ligands known to antagonize TRPV1 had different effects on thermoregulation (e.g., hyperthermia, hypothermia, or no effect) (181). In fact, TRPV1 can be manipulated in such a way, by action at different domains, to eliminate some functions of the TRPV1 channels without affecting others. For example, some antagonists block activation by capsaicin and high temperatures but not activation by low pH (182), and other antagonists block activation by capsaicin but not the activation by high temperature (183). However, this raises further questions on whether, for example, the observed effects are cell type specific. Again, this highlights the lack of fundamental knowledge on the role of TRPV1 in energy homeostasis and therefore the current challenges of targeting TRPV1 for the treatment of obesity.

## Conclusion

TRPV1 appears to be involved in energy homeostasis at a number of levels. In the periphery, TRPV1 activation or inhibition can have an impact of appetite and food intake through the control of appetite hormone levels or via the modulation of gastrointestinal vagal afferents, important for determining meal size and meal duration. In addition, TRPV1 plays a role in energy expenditure via heat production, either via direct thermogenesis or as a compensatory mechanism in response to TRPV1 induced heat-loss. Dietary supplementation with TRPV1 analogs, such as capsaicin, has yielded conflicting results with some studies demonstrating a decrease in food intake and increase in energy expenditure and others indicating the converse. This is probably reflective of the involvement of TRPV1 in a multitude of processes regulating food intake and energy expenditure. The story is complicated further by the interaction TRPV1 has with other systems involved in energy homeostasis, such as the endocannabinoid system. In addition, TRPV1 appears to be dysregulated in obesity, possibly due to alterations in the interaction with other systems. Therefore, although it is clear that TRPV1 plays a role in energy homeostasis without improved knowledge of the fundamental physiological mechanisms involved and the interactions with other systems it is impossible to target this system for the treatment of obesity, the maintenance of weight loss and the metabolic diseases associated with obesity.

## Author contributions

SC wrote the review under the supervision of GW and AP. HL edited the article and along with GW and AP provided assistance with the structure of the review.

### Conflict of interest statement

The authors declare that the research was conducted in the absence of any commercial or financial relationships that could be construed as a potential conflict of interest.

## Abbreviations

AC: adenylyl cyclase
AEA: anandamide
AMPK: adenosine monophosphate kinase
BAT: brown adipose tissue
BGL: blood glucose level
BMP: bone morphogenic protein
CAMK: calmodulin dependent kinase
CB: cannabinoid
DMV: dorsal motor nucleus of the vagus
GI: gastrointestinal
GLP: glucagon like peptide
PGC: PPAR gamma coactivator
PI3K: phosphatidylinositol 4-5 bisphosphate 3-kinase
PIP2, phosphatidylinositol-4: 5-bisphosphate
PKA: protein kinase A
PKC: protein kinase C
PLC: phospholipase C
PPAR: peroxisome proliferator activated receptor
PRDM: positive regulatory domain
SIRT: sirtuin
SNS: somatic nervous system
TRPV: transient receptor potential vanilloid
UCP: uncoupling protein
WAT: white adipose tissue.
